# Family history–based colorectal cancer screening in Australia: A modelling study of the costs, benefits, and harms of different participation scenarios

**DOI:** 10.1371/journal.pmed.1002630

**Published:** 2018-08-16

**Authors:** Mary Dillon, Louisa Flander, Daniel D. Buchanan, Finlay A. Macrae, Jon D. Emery, Ingrid M. Winship, Alex Boussioutas, Graham G. Giles, John L. Hopper, Mark A. Jenkins, Driss Ait Ouakrim

**Affiliations:** 1 Centre for Epidemiology and Biostatistics, Melbourne School of Population and Global Health, The University of Melbourne, Parkville, Victoria, Australia; 2 Systems Analysis Laboratory, Department of Mathematics and System Analysis, Aalto University, Aalto, Finland; 3 Colorectal Oncogenomics Group, Department of Clinical Pathology, The University of Melbourne, Parkville, Victoria, Australia; 4 Colorectal Medicine and Genetics, and the University of Melbourne Department of Medicine, The Royal Melbourne Hospital, Parkville, Victoria, Australia; 5 Department of General Practice and Centre for Cancer Research, University of Melbourne, Melbourne, Australia; 6 Genomic Medicine and the University of Melbourne Department of Medicine, The Royal Melbourne Hospital, Parkville, Victoria, Australia; 7 Peter MacCallum Cancer Centre, Melbourne, Australia; 8 Department of Medicine, The Royal Melbourne Hospital, Faculty of Medicine, Dentistry and Health Sciences, The University of Melbourne, Parkville, Victoria, Australia; 9 Cancer Epidemiology and Intelligence Division, Cancer Council Victoria, Melbourne, Australia; 10 Department of Epidemiology and Institute of Health and Environment, School of Public Health, Seoul National University, Seoul, Korea; University of Pittsburgh, UNITED STATES

## Abstract

**Background:**

The Australian National Bowel Cancer Screening Programme (NBCSP) was introduced in 2006. When fully implemented, the programme will invite people aged 50 to 74 to complete an immunochemical faecal occult blood test (iFOBT) every 2 years.

**Methods and findings:**

To investigate colorectal cancer (CRC) screening occurring outside of the NBCSP, we classified participants (*n* = 2,480) in the Australasian Colorectal Cancer Family Registry (ACCFR) into 3 risk categories (average, moderately increased, and potentially high) based on CRC family history and assessed their screening practices according to national guidelines. We developed a microsimulation to compare hypothetical screening scenarios (70% and 100% uptake) to current participation levels (baseline) and evaluated clinical outcomes and cost for each risk category. The 2 main limitations of this study are as follows: first, the fact that our cost-effectiveness analysis was performed from a third-party payer perspective, which does not include indirect costs and results in overestimated cost-effectiveness ratios, and second, that our natural history model of CRC does not include polyp sojourn time, which determines the rate of cancerous transformation.

Screening uptake was low across all family history risk categories (64%–56% reported no screening). For participants at average risk, 18% reported overscreening, while 37% of those in the highest risk categories screened according to guidelines. Higher screening levels would substantially reduce CRC mortality across all risk categories (95 to 305 fewer deaths per 100,000 persons in the 70% scenario versus baseline). For those at average risk, a fully implemented NBCSP represented the most cost-effective approach to prevent CRC deaths (AUS$13,000–16,000 per quality-adjusted life year [QALY]). For those at moderately increased risk, higher adherence to recommended screening was also highly cost-effective (AUS$19,000–24,000 per QALY).

**Conclusion:**

Investing in public health strategies to increase adherence to appropriate CRC screening will save lives and deliver high value for money.

## Abstract 2

Driss Ait Ouakrim and colleagues present a microsimulation to compare hypothetical increased CRC screening scenarios to current participation levels in Australia, and evaluate clinical outcomes and costs for each risk category.

## Author summary

**Why was this study done?:**

- Australia has one of the highest incidence rates of colorectal cancer (CRC) in the world. A population-based National Bowel Screening Programme (NBCSP) has been being progressively rolled out since 2006 (until 2020), but only 39% of its targeted population agree to participate.
- We have a limited understanding of the screening activity taking place outside of the current national programme—particularly for people at high risk of CRC due to family history.
- There is also a lack of empirical evidence on the costs and effects associated with different colorectal screening practices for different familial risk categories in the Australian population.

**What did the researchers do and find?:**

- We investigated the current CRC screening practices taking place outside of the existing screening programme, checked their consistency with the national guidelines on family history, and modelled the potential health benefits and harms as well as the economic impact associated with different screening behaviours.
- Our analysis showed that absence of, or inappropriate, screening concerns the vast majority of the population in Australia—including people at high risk of CRC due to their family history.
- A fully implemented NBCSP would be both the cheapest and most effective approach to prevent death from CRC in the general population compared with colonoscopy-based screening.

**What do these findings mean?:**

- The microsimulation model showed that higher screening levels would substantially reduce CRC mortality across all risk categories.
- Given the high health and economic burden of CRC in Australia and most industrialised countries—and the commonly low screening uptake seen in the populations of those countries—our findings highlight large health and economic benefits that would justify substantial investment in public health strategies to increase adherence to appropriate CRC screening.

## Introduction

Australia has one of the highest incidences of colorectal cancer (CRC) in the world [1]. CRC is currently the second most common malignancy diagnosed in Australians, accounting for 13.4% of all new cancer diagnoses and causing 8.7% of all cancer-related deaths [2]. These figures have remained stable over the last 30 years in Australia. But with a growing and aging population and escalating cost of new therapeutics, the cost of treating CRC has been rapidly increasing and was estimated at $1.2 billion per year in 2011, a 4-fold increase from 2001 [3].

Several randomised controlled trials have demonstrated the effectiveness of regular screening using faecal occult blood testing in reducing CRC incidence and mortality [4]. In 2006, the Australian federal government introduced a National Bowel Cancer Screening Programme (NBCSP), which, when fully implemented, will invite people aged 50 to 74 years to complete an immunochemical faecal occult blood test (iFOBT) every 2 years, free of charge. In its current form, the NBCSP is limited to those turning 50, 55, 60, 64, 65, 70, 72, or 74 years of age. The complete biennial roll-out of the programme is expected in 2020.

Alongside the NBCSP, there are also national CRC screening guidelines published by the National Health and Medical Research Council (NHMRC) [5]. These are addressed to the entire Australian population, as they take into account both age, personal history of colorectal adenomas and cancer, and family history of CRC to stratify people into different risk categories and provide specific screening recommendations for each (Table 1).

**Table 1:** NHMRC CRC familial risk categories and screening recommendations (2005).

Risk categories	Category 1: At or slightly above average risk	Category 2: Moderately increased risk	Category 3: Potentially high risk
Definition of family history	• No personal history of CRC, advanced adenoma, or chronic ulcerative colitis • No confirmed close family history of CRC • One FDR or SDR with CRC diagnosed at age 55 or older • Two FDRs or SDRs diagnosed with CRC at age 55 or older but on different sides of the family	• One FDR with CRC diagnosed before age 55 • Two FDRs or 1 FDR and 1 SDR on the same side of the family with CRC diagnosed at any age	• Three or more FDRs or SDRs on the same side of the family diagnosed with CRC • Two or more FDRs or SDRs on the same side of the family diagnosed with CRC plus any of the following features: (multiple CRCs in family member, CRC before age 50, family member with an HPNCC-related cancer)
Screening recommendations	• iFOBT every 2 years from age 50 or • Flexible sigmoidoscopy every 5 years	• Colonoscopy every 5 years from age 50+ or 10 years younger than the age of first CRC in the family, whichever comes first	• Colonoscopy every 1 or 2 years from age 25, or 5 years earlier than the youngest diagnosis in the family

Abbreviations: CRC, colorectal cancer; FDR, first-degree relative; HNPCC, hereditary nonpolyposis colorectal cancer; iFOBT, immunochemical faecal occult blood test; NHMRC, National Health and Medical Research Council; SDR, second-degree relative.

Data on CRC screening participation in Australia are scarce [6]. The latest monitoring report from the NBCSP showed that only 39% of the people invited to screen completed the iFOBT kit [2]. The reported NBCSP participation does not take into account the large amount of screening occurring outside of the programme (i.e., opportunistic screening) [7,8]. We currently have a limited understanding of this screening activity, in particular with respect to family history.

There is also a lack of empirical evidence on the costs and effects associated with different CRC screening practices in Australia. We know, for example, that the Australian government spent AUS$51.8 million on the NBCSP in 2014–2015 [2], but this amount does not include CRC screening outside the programme. The most extensive study to date on the cost-effectiveness of screening is an assessment of the NBCSP that estimated the number of CRC cases and deaths that could be avoided, and the associated costs to the health budget, if screening participation in the NBSCP was increased [9]. However, this study was unable to assess screening by family history.

Our aim was to investigate current CRC screening practices in the Australian population within and outside of the NBCSP, check their consistency with the existing NHMRC guidelines on family history, and evaluate the economic and health benefits and harms potentially associated with these practices.

## Material and methods

### Study sample

We used data provided by participants in the Australasian Colorectal Cancer Family Registry (ACCFR), a large population-based family cohort study designed to address specific research questions on CRC aetiology and prevention. Details of the methodology and data collected by the ACCFR have been described elsewhere [10,11] and are available at http://coloncfr.org. Briefly, participants were recruited between 1997 and 2012 via population-based case probands and population-based control probands. Epidemiologic and demographic information was collected using in-person interviews, telephone interviews, or mailed questionnaires at the time of recruitment (available at http://coloncfr.org/questionnaires-forms). Case probands were residents of the Melbourne metropolitan area between 1997 and 2001 who were diagnosed when aged between 18 and 59 years with an incident first primary adenocarcinoma of the colorectum identified by the Victorian Cancer Registry. People who had a previous diagnosis of CRC or familial adenomatous polyposis (FAP) were excluded. Attempts were made to recruit adult first- and second-degree relatives (parents, siblings, offspring, aunts, uncles, and grandparents) of all case probands as well as their spouses or partners. Population control probands—frequency-matched to the age and sex of the case probands and registered as living in the Melbourne metropolitan—were identified from the federal electoral register (registering to vote is compulsory for all Australians 18 years old and older). Similarly to the case probands, control probands were asked permission to contact their first- and second-degree relatives regarding participation in the ACCFR. Attempts were made to follow-up all participants every 4 to 5 years to update their screening history, cancer diagnoses, and family history. For this analysis, we included all ACCFR participants interviewed by follow-up questionnaire between 2009 and 2012 (the NBCSP was then in its second stage of roll-out, inviting 50-, 55-, and 65-year-olds to participate) with no previous CRC diagnosis and who completed family history and a risk factor questionnaire including items on screening over the previous 5 years—2,714 participants in total (see S1 Fig).

### CRC risk categories and screening participation

Participants were classified according to existing NHMRC guidelines into 3 CRC risk categories based on their age, family history of CRC, and age at diagnosis of affected relatives (see Table 1 for description and definition of categories). Screeners were defined as participants who reported having undergone iFOBT, flexible sigmoidoscopy, or colonoscopy either as a regular check-up or because of their family history of CRC. Nonscreeners were those who did not report any screening or who had undergone 1 of the 3 procedures, but only to investigate a new problem or as follow-up of a previous problem. They were therefore considered either diagnostic procedures or surveillance. We deemed screening as appropriate if the screening regimen reported by the participants was consistent—in term of age, frequency, and modality—with the NHMRC guideline recommendations for each CRC risk category (Table 1). Underscreening was defined as screening at a lower frequency than recommended or with a stool-based test instead of colonoscopy when the latter is recommended based on the individual’s risk category. Overscreening was defined as screening at a younger age or at a higher frequency than recommended, or with colonoscopy (at least 1 in the last 10 years) without fulfilling the age and family history criteria defined by the guidelines to warrant screening with such a procedure. Proportions of participants in each risk category were calculated separately, based on the screening practices reported by the participant and the risk categories to which they were allocated. We refer the reader to our previous reports for further details on the definitions of participants’ screening behaviour and risk categorisation [7,8].

## Economic model

**Fig 1:**
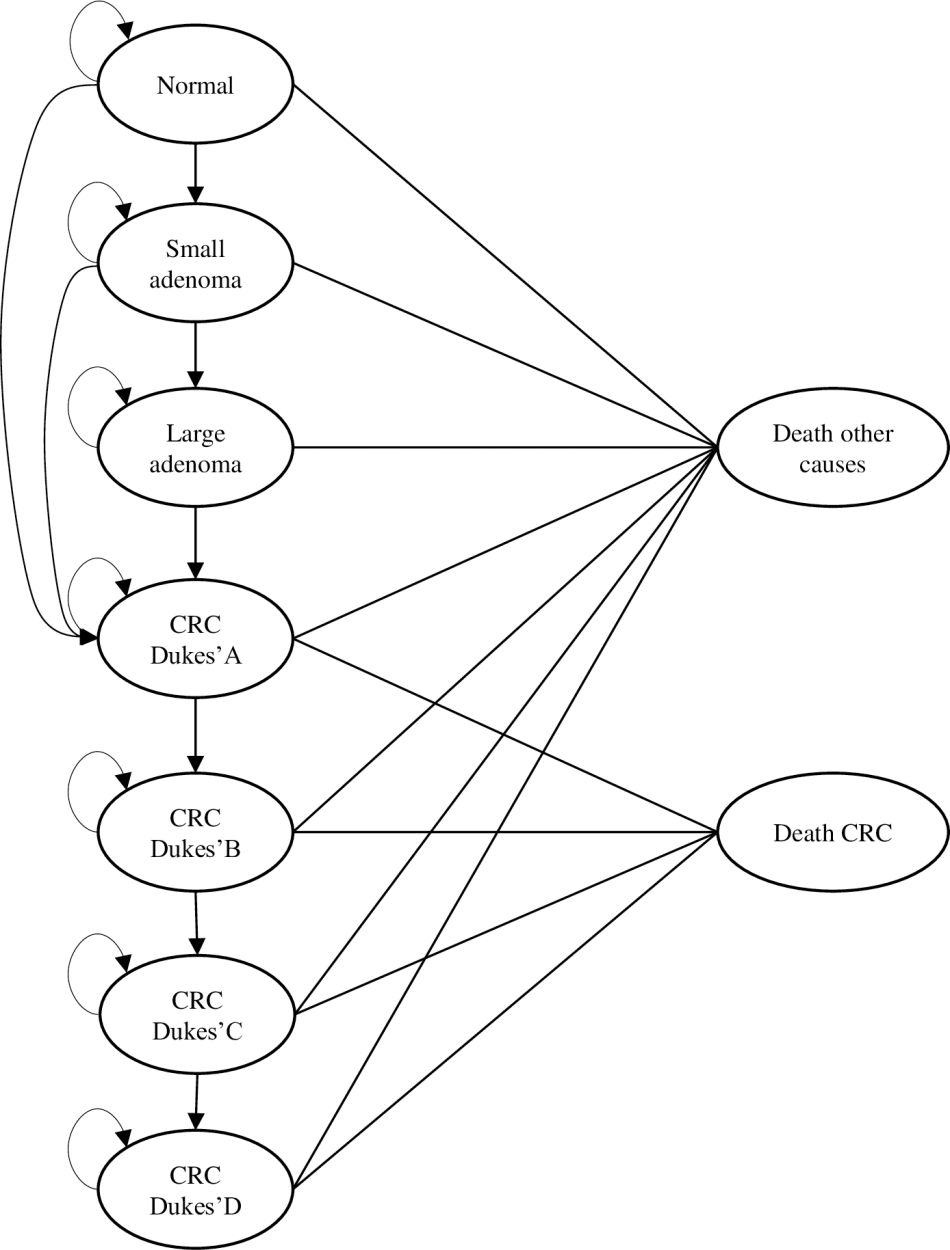
Structure of the microsimulation model. Note: all states (apart from death, which is absorbing) are transient, which enables individuals to move to other states by the predetermined state transition probabilities or remain in the same state. CRC, colorectal cancer.

We developed a Markov microsimulation to assess the expected clinical and economic impact of appropriate, under-, and overscreening. The analysis extends a model previously developed by Ait Ouakrim and colleagues [12] that simulates a hypothetical population of 100,000 individuals and their progression through 9 mutually exclusive health states representing CRC progression—from normal bowel to adenoma, CRC bowel states, and death from CRC or other causes. Given that only 70% to 80% of CRCs develop via the transformation of an adenomatous polyp [13,14] commonly referred to as the adenoma–carcinoma sequence, the model allows for a small proportion of CRCs to develop via an alternative pathway. Therefore, individuals in the health states “normal bowel” and “small adenoma” can progress to CRC Dukes A without having to pass through the modelled adenoma–carcinoma pathway (see Fig 1). Movement between states was determined by state transition probabilities identified in the literature.

### Model parameters

In the model, all individuals enter at age 25 years and are able to exit at death or age 100 years, whichever occurs first. Age-specific prevalence and incidence probabilities of adenoma and CRC were obtained from the health economic review published by Bishop and colleagues [15] with updated calculated age-standardised incidence rates [2,16] (model parameters including initial-state probabilities, utilities, and costs are provided in S1 Table). To account for the higher risk of CRC experienced by individuals in risk category 2, we multiplied the population age-specific incidence of adenoma and CRC by a factor of 4 (relative risk [RR] = 4) on the basis of the current NHMRC criteria of 3- to 6-fold increased risk for this subgroup of the population compared with the average risk subgroup of the population (i.e., risk category 1) [5]. Similarly, for individuals in category 2, we multiplied the estimates of age-specific incidence of small adenomas, large adenomas, and CRC at different stages for the general population by 4 to calculate the age-specific transition probabilities. We followed the same approach for individuals in category 3 by multiplying the relevant parameters by a factor of 15 (see S2–S4 Tables).

### Screening scenarios

To assess the impact of current and hypothetical screening participation in the population, we conducted 10 experiments altogether under the 3 defined risk categories:

Category 1: At or slightly above average risk

- Actual screening—based on ACCFR analysis (baseline)
- 39% participation in full NBCSP only—based on current NBCSP data (current)
- 70% participation in full NBCSP only—(aspirational)
- 100% participation in full NBCSP only—(complete)

The NBCSP scenarios above assumed that only biennial iFOBT screening between ages 50 and 74 was occurring, i.e., no one was undergoing colonoscopy screening.

Category 2: Moderately increased risk

- Actual screening—based on ACCFR analysis (baseline)
- 70% compliance with NHMRC guidelines—(aspirational)
- 100% compliance with NHMRC guidelines—(complete)

Category 3: Potentially high risk

- Actual screening—based on ACCFR analysis (baseline)
- 70% compliance with NHMRC guidelines—(aspirational)
- 100% compliance with NHMRC guidelines—(complete)

Here, the actual screening strategies were defined as the current screening practices in Australia—which includes current level of adherence to the NHRMC recommendations based on the analysis of the ACCFR dataset—whereas the full NBCSP strategies were based on the screening eligibility criteria of the NBCSP once fully implemented (i.e., biennial iFOBT screening from age 50 years to 74 years).

### Costs

The analysis of the model was undertaken from the perspective of a third-party payer in the public health system and included only direct costs to the government. Each cost parameter was presented in 2016 AUS$ prices. For the cost parameters requiring correction for inflation, the ABS Consumer Price Index Inflation Calculator [16] was used. The cost of a colonoscopy was calculated using the reported colonoscopy cost in the Australian Public Hospital Cost Report 2013–2014 [17], weighted due to a 1.63% chance of an unplanned hospital visit within 7 days of the procedure, as reported by Ranasinghe and colleagues [18], and inflated to 2016 prices. The cost of a colonoscopy with polypectomy was calculated by using the aforementioned colonoscopy cost and increasing it by 40% due to the difference between the Medicare Benefits Scheme (MBS) item 32088 (without polypectomy) and item 32089 (with polypectomy) [19]. We applied a 5% annual discount to all costs and utilities within the model, in keeping with Australian health technology assessment agencies.

### Model outcomes

The primary outcomes of the model were the expected total costs, quality-adjusted life years (QALYs), and the incremental cost-effectiveness ratio (ICER) of each screening scenario. Secondary outcomes were the total number of CRC-attributed deaths, colonoscopy procedures, and associated adverse events (i.e., bleeds, perforations, and deaths due to colonoscopy).

### Sensitivity analysis

To assess the robustness of the model, one-way sensitivity analysis was conducted by analysing the impact of the uncertainty that surrounds the values of the following variables: cost values, utility values, and RR factors of CRC. To identify which variables were most sensitive to change, tornado diagrams for each risk level were produced for cost and utility values. Each cost and utility variable was varied by 20%, apart from the utilities that were capped at a value of 1 (perfect health). The RR was varied according to the NHMRC criteria on increased risk in each risk category compared with the population risk.

Further information on the planning and design of this study is provided in S1 Text.

## Results

### Screening participation

Table 2 presents the CRC screening participation of ACCFR participants by age group and for each risk category. Of the 3,241 participants interviewed between 2009 and 2012, 2,480 had no previous diagnosis of CRC and had complete data on screening participation and were, therefore, included in the analysis. Of these, 954 were categorised as “at or slightly above average risk” (risk category 1), 1,006 as “at moderately increased risk” (category 2), and 520 as “at potentially high risk” (category 3) (see S1 Fig). Participation varied by age group and by risk category. Overall, for people in risk category 1, 64% reported no screening, 4% reported some screening but less than recommended, 14% reported appropriate screening, and 18% reported more than recommended screening. For people in risk category 2, 62% reported no screening, 1% reported some screening but less than recommended, 37% reported appropriate screening, and 0% reported more than recommended screening. For people in category 3, 56% reported no screening, 7% reported some screening but less than recommended, 37% reported appropriate screening, and 0% reported more than recommended screening.

**Table 2:** CRC screening participation in the ACCFR by age and risk category.

Age category	18-39 years			40-49 years			50-59 years			60-69 years			≥70 years		
	n	%	95% CI	n	%	95% CI	n	%	95% CI	n	%	95% CI	n	%	95% CI
**Risk category 1 (N=954)** *At or slightly above average risk*															
Never screened	*111*	*98*.*3*	*(93-99*.*5)*	*185*	*93*.*9*	*(89*.*5-96*.*5)*	108	63.1	(55.6-70.0)	194	64.2	(58.6-69.4)	113	66.1	(58.6-72.8)
Some screening	na			na			11	6.4	(3.5-11.2)	12	3.9	(2.2-6.8)	4	2.4	(0.1-6.0)
Appropriate screening	na			na			25	14.6	(10.0-20.7)	47	15.5	(11.8-20.1)	17	9.9	(6.2-15.4)
Over-screening	2	1.7	(0.4-6.8)	12	6.1	(3.4-10.4)	27	15.9	(11.0-22.0)	49	16.4	(12.4-20.8)	37	21.6	(16.0-28.4)
**Risk category 2 (N=1006)** *Moderately increased risk*															
Never screened	*213*	*93*.*1*	*(88*.*8-95*.*6)*	*118*	*60*.*5*	*(53*.*4-67*.*1)*	119	50.2	(43.8-56.5)	78	40.6	(33.8-47.7)	101	66.0	(58.1-73.1)
Some screening	na			na			3	1.2	(0.4-3.8)	2	1	(0.2-4.0)	1	0.7	(0.09-4.5))
Appropriate screening	na			na			115	48.6	(42.2-54.8)	112	58.4	(51.2-65.1)	51	33.3	(26.3-41.2)
Over-screening	16	6.9	(4.3-11.1)	77	39.5	(32.8-46.5)	0			0			0		
**Risk category 3 (N=520)** *Potentially high risk*															
Never screened	56	91.8	(81.1-96.5)	75	65.2	(56.0-73.3)	69	50.3	(42.0-58.6)	50	41.6	(33.1-50.7)	39	44.8	(34.6-55.4)
Some screening	1	1.6	(0.2-10.9)	8	6.9	(3.5-13.3)	3	2.19	(0.7-6.6)	13	10.8	(6.3-17.8)	13	14.9	(8.8-24.1)
Appropriate screening	4	6.5	(2.4-16.3)	32	27.8	(20.3-36.7)	65	47.4	(39.1-55.8)	57	47.5	(38.6-56.4)	35	40.2	(30.4-50.9)
Over-screening	0			0			0			0			0		

CI: confidence interval

### Economic evaluation

#### Risk category 1

**Fig 2:**
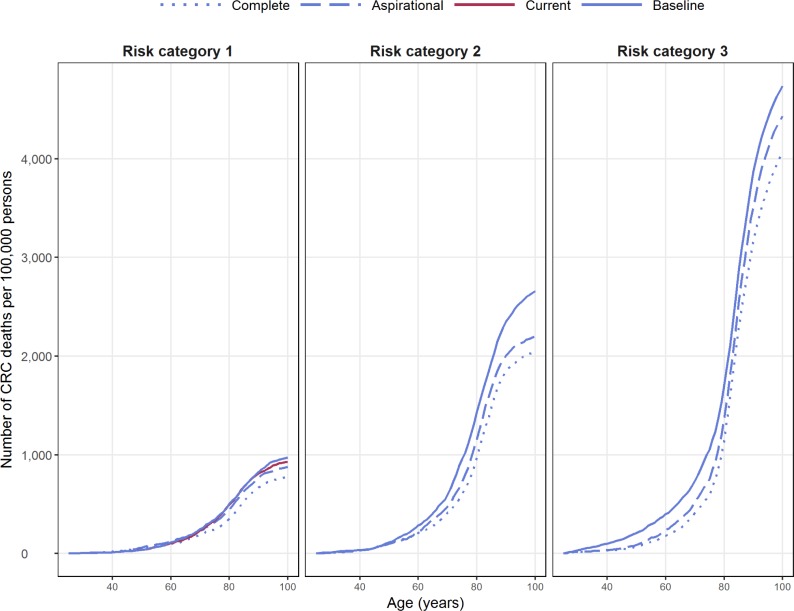
Deaths attributable to CRC for different screening scenarios. CRC, colorectal cancer.

For this subgroup of the population at average risk, the baseline screening scenario (actual screening) was associated with the highest incidence of CRC deaths over the simulated 75 years of their lives (972 deaths per 100,000 persons), followed by the current scenario of NBSCP only (preventing 42 fewer deaths per 100,000 persons) (Table 3, Fig 2). Under the aspirational (70% appropriate NBCSP screening) scenario, the average number of CRC deaths per 100,000 persons was reduced compared with both the baseline and current scenarios estimate (Table 3), with an estimated cost per death prevented of $78,000. Larger reductions in CRC mortality occurred under the complete (100% appropriate NBCSP screening) scenario when compared with the baseline, with an estimated cost per death prevented of $120,000.

**Table 3:** Clinical and cost outcomes from the microsimulation for each CRC risk category.

Screening scenario	Total number of CRC-attributed deaths per 100,000	Total number of colonoscopies per 100,000	Total number of deaths due to colonoscopy complications per 100,000	Average lifetime cost*		Average lifetime effectiveness		ICER (AU$/QALY)
				AU$/person	95% UI	QALYs/person	95% UI	
Risk category 1^**^								
Baseline	972	130,373	22	473.11	(470.71–475.21)	18.832	(18.821–18.843)	8,523
Current	−42	−67,022	−18	−143.73	(326.78–331.98)	−0.017	(18.804–18.826)	-
Aspirational	−95	−8,458	−9	+73.84	(543.15–550.75)	−0.001	(18.820–18.842)	13,219
Complete	−192	+26,761	−10	+229.55	(698.46–706.86)	+0.006	(18.827–18.849)	16,110
**Risk category 2**								
Baseline	2,661	298,797	14	1,146.37	(1,141.97–1,150.77)	18.673	(18.662–18.684)	-
Aspirational	−459	+100,053	+15	+370.30	(1,512.17–1,521.17)	+0.019	(18.681–18.703)	19,410
Complete	−622	+237,384	+35	+900.32	(2,042.69–2,050.69)	+0.037	(18.699–18.721)	24,326
**Risk category 3**								
Baseline	4,739	1,583,263	140	7,922.05	(7,904.05–7,940.05)	18.450	(18.439–18.461)	-
Aspirational	−305	+1.55 million	+96	+15,870.15	(23,767.20–23,817.20)	+0.112	(18.551–18.573)	142,156
Complete	−686	+2.61 million	+190	+24,682.09	(32,578.14–32,630.14)	+0.130	(18.569–18.591)	190,103

*Average lifetime cost and ICERs in US$ are available in S5 Table.

**For category 1, the current scenario is taken as the base for calculating the ICERs by increasing effectiveness.

Abbreviations: CRC, colorectal cancer; ICER, incremental cost-effectiveness ratio; QALY, quality-adjusted life year; UI, uncertainty interval.

**Fig 3:**
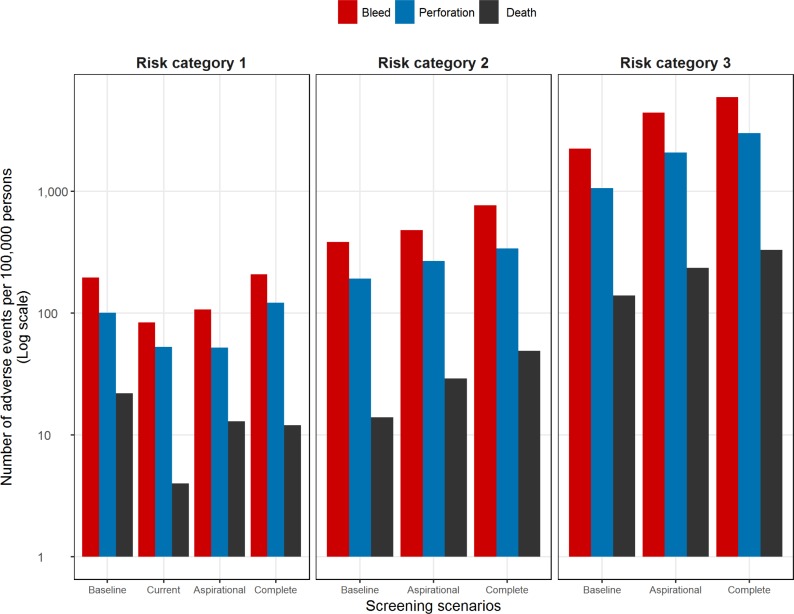
Number of adverse events associated with colonoscopy under different screening scenarios.

**Fig 4:**
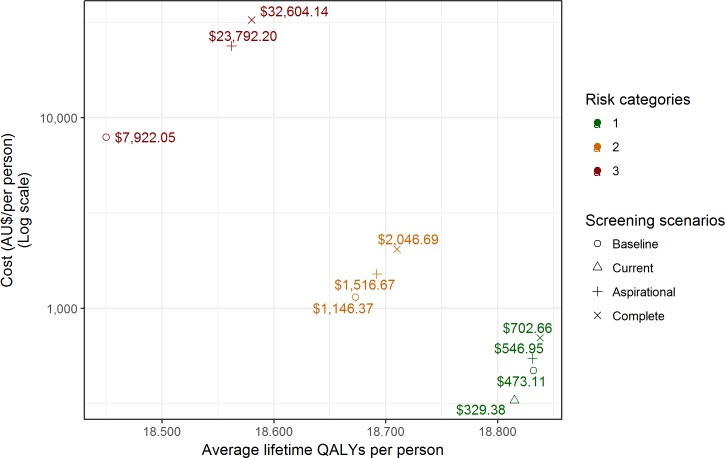
Cost and QALYs associated with different screening scenarios. QALY, quality-adjusted life year.

In terms of adverse events, the baseline scenario resulted in 130,000 colonoscopy procedures performed (per 100,000 persons), resulting in additional perforations (n = 101), bleeds (n = 196), and deaths (n = 22) compared with the current scenario. In the aspirational scenario, the number of adverse events decreased compared with the baseline as a result of applying appropriate screening only and the absence of overscreening. However, the number of colonoscopies, and hence the number of adverse events, increased compared with the current scenario (Table 3, Fig 3). The complete scenario resulted in the highest number of colonoscopies performed with, on average, 1.6 colonoscopies per person over their lifetime. The baseline scenario was associated with a total lifetime cost of AUS$47.3 million and total lifetime effectiveness of 1.88 million QALYs per 100,000 persons. In comparison, the lifetime cost of the current scenario was estimated to be AUS$32.9 million per 100,000 persons and resulted in 1,686 fewer total QALYs per 100,000, resulting in a baseline ICER of AUS$8,523 (Table 3, Fig 4).

#### Risk category 2

For risk category 2, only the scenarios modelled by the NHMRC guidelines were applied. The baseline scenario was associated with the highest number of CRC deaths (2,661 per 100,000); in comparison, the aspirational scenario was associated with 459 fewer CRC deaths, and the complete-compliance scenario was associated with 622 fewer (Table 3, Fig 2). Under the increased-compliance screening scenarios, the number of CRC-attributed deaths was reduced compared with the baseline estimates, resulting in a cost per death prevented of $81,000 and $145,000 for the aspirational and the complete scenarios, respectively.

In terms of adverse events due to colonoscopy, the baseline scenario was associated with 298,797 colonoscopies per 100,000 individuals, resulting in 198 complications per 100,000 procedures (Fig 3). Under the aspirational and complete screening scenarios, the number of adverse events increased along with the number of colonoscopies performed. The total number of colonoscopies increased by 100,000 and 237,000 per 100,000 for the aspirational and complete scenarios, respectively, compared with the baseline scenario (Table 3).

The baseline scenario incurs a total lifetime cost of AUS$114.6 million and 1.87 million QALYs per 100,000 persons. In comparison, the aspirational and complete scenarios led to additional total lifetime cost and total lifetime effectiveness, resulting in ICERs of AUS$19,410 and AUS$24,326, respectively (Table 3, Fig 4).

#### Risk category 3

For individuals at potentially high risk of CRC—risk category 3—the baseline scenario was associated with the highest number of CRC deaths with 4,739 deaths per 100,000 persons, representing 5.1% of the total number of deaths in the simulation for this risk category (Fig 2). In comparison, the aspirational compliance with the NHRMC guidelines scenario resulted in 305 fewer CRC-attributed deaths (Table 3, Fig 2) than the baseline (a 0.4% reduction), with a cost of AUS$5.2 million per CRC death prevented. Under the complete scenario, the number of CRC deaths reduced even further (a total of 0.7% reduction from the baseline) and resulted in a cost of AUS$3.6 million per death prevented.

The number of adverse events due to colonoscopy in the baseline scenario was substantially different from the increased-compliance scenarios (Fig 3). These adverse events were the result of 1.58 million colonoscopies performed over the lifetime of 100,000 persons in the baseline scenario, 3.13 million in the aspirational scenario, and 4.19 million in the complete scenario (Table 3). This increase was reflected in the number of adverse events, including death attributed to colonoscopy. It is important to note, however, that the increases in death due to colonoscopy do not outweigh the additional lives saved from death due to CRC.

The baseline scenario was associated with a total lifetime cost of AUS$792 million and 1.85 million QALYs per 100,000 persons; this led to an ICER of AUS$142,000 for the aspirational scenario and AUS$190,000 for the complete scenario (Table 3, Fig 4).

### Sensitivity analysis

The results of the sensitivity analysis illustrate the influence that the variables have on the baseline results and, hence, which contribute the most variability to the cost and/or the effectiveness (see S6–S8 Tables). For each of the risk categories, the cost variable for colonoscopy without polypectomy was the most sensitive to change, with the later stages of CRC (Dukes B–D) annual treatment costs having little to no effect on the average lifetime cost (see S2–S4 Figs). Risk categories 2 and 3 were more sensitive to the cost of a polypectomy than the average risk category. The effect of the sensitivity analysis for the utility values was most apparent for the normal bowel utility value, with it having the greatest effect on the average lifetime QALYs. In particular, the decrease of 20% in the normal bowel utility value (to 0.8) resulted in a reduction of effectiveness to around 15 QALYs per person for each risk category (see S5–S7 Figs). This is important to highlight because in the baseline scenario, the effectiveness is above 18 QALYs per person for each risk category. The small and large adenoma utility values were the next most sensitive in our model, but far less than for the normal bowel. Dukes A utility value had little effect on risk category 1, with only a slight effect on risk category 2 and 3. The later stages of Dukes CRC utility values had little to no effect on the sensitivity of the model. The results of the RR sensitivity analysis displayed what was expected in terms of cost and effectiveness. For each risk level, when the RR was modified, the cost remained stable, yet the effectiveness varied on the baseline results.

## Discussion

Our first objective was to investigate CRC screening practices in the Australian population and characterise them according to national screening guideline recommendations and CRC risk categories based on family history. Our results provide a complex picture of CRC screening in Australia. Absence of, or inappropriate, screening concerns the vast majority of the population. Of eligible people in risk category 1—being at or slightly above average risk, which is the case for 98% of the population—approximately two-thirds had never undergone a CRC screening test, while 10% to 15% had been screened according to guideline recommendations and 16% to 21% had been overscreened given their risk category (by undergoing colonoscopies). A total of 2% to 6% engaged in CRC screening but less often than recommended.

For those in risk category 2, approximately half of the eligible population screened according to the guidelines, both in terms of recommended frequency and procedure (colonoscopy), while the other half did not screen at all. Some overscreening occurred in the age categories under 50 years, particularly for those aged 40 to 49 years.

For people in risk category 3, the level of appropriate screening was between 40% and 47% for those aged over 50 years and only 28% for those aged 40 to 49 years. Most importantly, the vast majority of those not screening according to the guideline recommendations (i.e., colonoscopy every 1 to 2 years) reported no screening activity at all. This is particularly concerning given that people in this risk category are considered as being at potentially high risk of CRC, and particularly for early-onset CRC, which often results in more aggressive forms of CRC [20].

These results are consistent with our previous reports (based on surveys conducted between 1997 and 2001), which also identified important levels of underscreening or no screening across all risk categories, along with a substantial level of overscreening among people in the lowest risk level of CRC, who represent the largest segment of the Australian population [7,8]. The estimates reported in this study, however, provide a more accurate picture of the current CRC screening practices in Australia because they stem from population-based surveys conducted between 2009 and 2012. They are also consistent with the evolution of CRC screening policies in Australia and the continuous expansion of the NBCSP since its introduction in 2006. For example, the high level of appropriate screening seen in risk categories 2 and 3 (approximately 50%) certainly reflects increased awareness of CRC among physicians and the public. In comparison, in our previous analyses, only 6% of people in risk category 2 and 1% of those in risk category 3 reported appropriate screening. Better awareness of the disease is also likely to be reflected in the overscreening practices—through colonoscopy screening—reported by a substantial proportion of people in risk category 1. Overscreening might also be a result of the growth in the provision of colonoscopy procedures seen in Australia over recent years [21].

Our second objective was to model the long-term health and economic impact of the screening practices identified in our analysis of the ACCFR data and to estimate the effectiveness and cost-effectiveness of different current and hypothetical screening and participation scenarios for the 3 NHMRC-defined CRC risk guideline categories.

The microsimulation showed that the baseline screening scenario was the lowest performing approach because, although it resulted in a higher number of QALYs, it was also associated with the largest number of CRC-attributed deaths across all screening scenarios in risk category 1. This opportunistic screening as it currently occurs in Australia—i.e., approximately 60% of the population underscreening, 20% screening appropriately, and 20% overscreening with colonoscopy—comes with a high price tag (AUS$47 million lifetime cost per 100,000 persons) for those in risk category 1, along with a substantial number of adverse events and deaths associated with the high number of unwarranted colonoscopies.

Our modelling also showed that a fully implemented NBCSP would reduce the number of CRC deaths compared with the current screening practices of Australians in the first risk category (see Table 2 and Fig 2). According to our model, regardless of the level of screening participation in the population of category 1, a fully implemented NBCSP outperforms—in terms of CRC deaths prevented—a screening regimen based on 1 colonoscopy procedure every 10 years.

For risk categories 1 and 2, the aspirational and complete scenarios were associated with ICERs under AUS$25,000 per QALY, which is half the dollar amount per life year gained regarded as the upper limit of acceptable cost-effectiveness in the Australian health system [22]. For policy makers, this means that investing in public health strategies to increase adherence to appropriate CRC screening will deliver high value for money. It should be noted that our ICER estimates are likely to be substantially inflated as a result of the third-party payer perspective applied to the model. A broader approach—one that takes into account the indirect costs due to CRC and premature death, such as loss in productivity and labour supply—would likely reduce the ICERs and therefore increase the cost-effectiveness of the high screening uptake scenarios for all risk categories.

Our results highlight the benefits for increased screening participation and thus justify more action and sustained efforts from governments to improve adherence to CRC screening—particularly in Australia, where the NBCSP has never achieved a participation rate higher than 40% [23]. Nonadherence to guideline recommendations and low screening uptake in the population characterise the vast majority of existing screening initiatives worldwide and represent the main barrier to their full effectiveness in terms of averted CRC incidence and mortality. For example, most European countries with an established iFOBT-based screening programme report participation rates below 50% [24]. In the United States—where 65% of adults are compliant with CRC screening recommendations—studies have shown that a substantial percentage of CRC deaths are attributable to nonuse of screening [25,26]. Our modelling CRC screening practices in Australia, in line with evidence from other countries [27], provides estimates of the health and economic benefits forgone in a context of low CRC screening as well as of those that could result from higher levels of appropriate screening practices. The findings of this study are therefore relevant to all countries where CRC is a public health concern and population screening uptake low.

One of the main strengths of our study is the ability to present screening participation with respect to specific CRC risk levels defined by family history of cancer. This was possible because of our systematic data collection from all participants and systematic attempts to validate information provided by relatives in the ACCFR. The design of the ACCFR—enriched with people with a family history of CRC—allowed us to have enough participants in each CRC risk category, including for the ‘population risk’ category, and thus sufficiently precise parameter estimates to derive our analyses.

To our knowledge, this is the first study to assess the health and economic effect of opportunistic CRC screening (defined as our baseline scenarios based on the ACCFR analysis) across all family history risk categories in the population as well as the effects of the future, fully implemented NBCSP. Our aim was not to demonstrate the health and economic advantages of CRC screening because those advantages have been consistently shown by many studies in a variety of contexts [28–30]. We therefore deliberately did not consider a ‘no screening’ scenario in our analysis because it does not represent the current reality of CRC screening policy in Australia. Our goal was to assess how alternative screening scenarios and compliance levels might impact, positively or negatively, on CRC screening outcomes.

Our modelling has several limitations. We assumed that family history of CRC was known for all participants in the simulated population and did not include administrative costs associated with the implementation of a family history assessment. While we attempted to account for the higher incidence of adenomas observed in people with family history of CRC as a cause of a higher CRC incidence, we did not include in our modelling information on polyp sojourn time (i.e., preclinical phase), which determines the rate of cancerous transformation. Adenoma behaviour in those with family history is still not well characterised, particularly the malignancy transformation rate.

## Conclusion

This study provides a reference to which the performance of the national programme can be compared. A fully implemented NBCSP appears as both the cheapest and most effective approach to prevent death from CRC in the general population compared with colonoscopy-based screening. According to our model, this performance holds even with the 2017 NBCSP participation level, which is around 39%. Currently, opportunistic screening—i.e., screening outside of the existing organised programme—despite its high cost appears to be the most appropriate way to access screening for those at higher risk of CRC due to their family history as long as the specific needs of people in this category are not taken into account by the NBCSP. A programmatic approach to offering appropriate colonoscopy-based screening to the highest-risk group may provide further benefits in terms of cost and CRC deaths prevented.

## Supporting information

S1 Text Analysis plan. (DOCX)

S1 Fig ACCFR participant selection flowchart. ACCFR, Australasian Colorectal Cancer Family Registry.
(TIF)

S2 Fig Tornado diagram for cost sensitivity analysis—risk category 1. (TIF)

S3 Fig Tornado diagram for cost sensitivity analysis—risk category 2. (TIF)

S4 Fig Tornado diagram for cost sensitivity analysis—risk category 3. (TIF)

S5 Fig Tornado diagram for utility values sensitivity analysis—risk category 1. (TIF)

S6 Fig Tornado diagram for utility values sensitivity analysis—risk category 2. (TIF)

S7 Fig Tornado diagram for utility values sensitivity analysis—risk category 3. (TIF)

S1 Table Model parameters. (DOCX)

S2 Table Incremental incidence for risk category 1, by age group. (DOCX)

S3 Table Incremental incidence for risk category 2, by age group. (DOCX)

S4 Table Incremental incidence for risk category 3, by age group. (DOCX)

S5 Table Cost outcomes from the microsimulation expressed in US$. (DOCX)

S6 Table Sensitivity analysis of cost variables for all risk categories. (DOCX)

S7 Table Sensitivity analysis of utility values for all risk categories. (DOCX)

S8 Table Sensitivity analysis of RR factors for all strategies, and risk categories. (DOCX)

## Abbreviations

ACCFR: Australasian Colorectal Cancer Family Registry
CRC: colorectal cancer
FAP: familial adenomatous polyposis
HNPCC: hereditary nonpolyposis colorectal cancer
ICER: incremental cost-effectiveness ratio
iFOBT: immunochemical faecal occult blood test
MBS: Medicare Benefits Scheme
NBCSP: National Bowel Cancer Screening Programme
NHMRC: National Health and Medical Research Council
QALY: quality-adjusted life year
RR: relative risk
UI: uncertainty interval
