# Supplementary material for: Family history–based colorectal cancer screening in Australia: A modelling study of the costs, benefits, and harms of different participation scenarios
Source: PLoS Med. 2018 Aug 16;15(8):e1002630. doi: 10.1371/journal.pmed.1002630 (PMC6095490; doi:10.1371/journal.pmed.1002630)
Supplement: S1 Text — (DOCX) [file pmed.1002630.s001.docx]

# Analysis plan

This study was planned in 2014 and initially designed as an update of two previous papers [1,2] in which we investigated CRC screening practices for different family risk categories in the Australian population. One of the limitations of those studies was the fact that they were based on data collected between 1998 and 2001, which was prior to the substantive roll-out of the national colorectal cancer screening programme. Therefore, their results, in terms of screening uptake, were likely to under estimate the current level of colorectal cancer screening participation. The aim of the new study was to perform similar analyses using data collected between 2009 and 2012, which would better reflect current screening behaviour and any potential improvements in colorectal cancer screening awareness in the population – due, for example, to the introduction of the National Bowel Cancer Screening Program in 2006.

Another limitation was lack of cost-effectiveness analysis and therefore inability to provide opportunity costs associated with low screening participation. We therefore decided to alter the design of the study by adding a substantial health economic component to the initial analysis. The rational for this change was, to go beyond a simple descriptive analysis and to provide policy makers with estimates of the opportunity cost associated with inappropriate colorectal cancer screening and an under-utilised national screening programme, for the Australian health system. We anticipate that this approach might facilitate the translation of our findings and their integration into future decisions on colorectal cancer screening policy.

References

1. Ait Ouakrim D, Boussioutas A, Lockett T, Winship I, Giles GG, Flander LB, et al. Screening practices of unaffected people at familial risk of colorectal cancer. Cancer Prev Res (Phila). 2012;5(2):240-7. Epub 2011/10/28. doi: 10.1158/1940-6207.CAPR-11-0229. PubMed PMID: 22030089; PubMed Central PMCID: PMC3273548.

2. Ait Ouakrim D, Lockett T, Boussioutas A, Keogh L, Flander LB, Winship I, et al. Screening practices of Australian men and women categorized as "at or slightly above average risk" of colorectal cancer. Cancer Causes Control. 2012. Epub 2012/09/27. doi: 10.1007/s10552-012-0067-y. PubMed PMID: 23011536.
