# Supplementary figures and images for: Family history–based colorectal cancer screening in Australia: A modelling study of the costs, benefits, and harms of different participation scenarios
Source: PLoS Med. 2018 Aug 16;15(8):e1002630. doi: 10.1371/journal.pmed.1002630 (PMC6095490; doi:10.1371/journal.pmed.1002630)

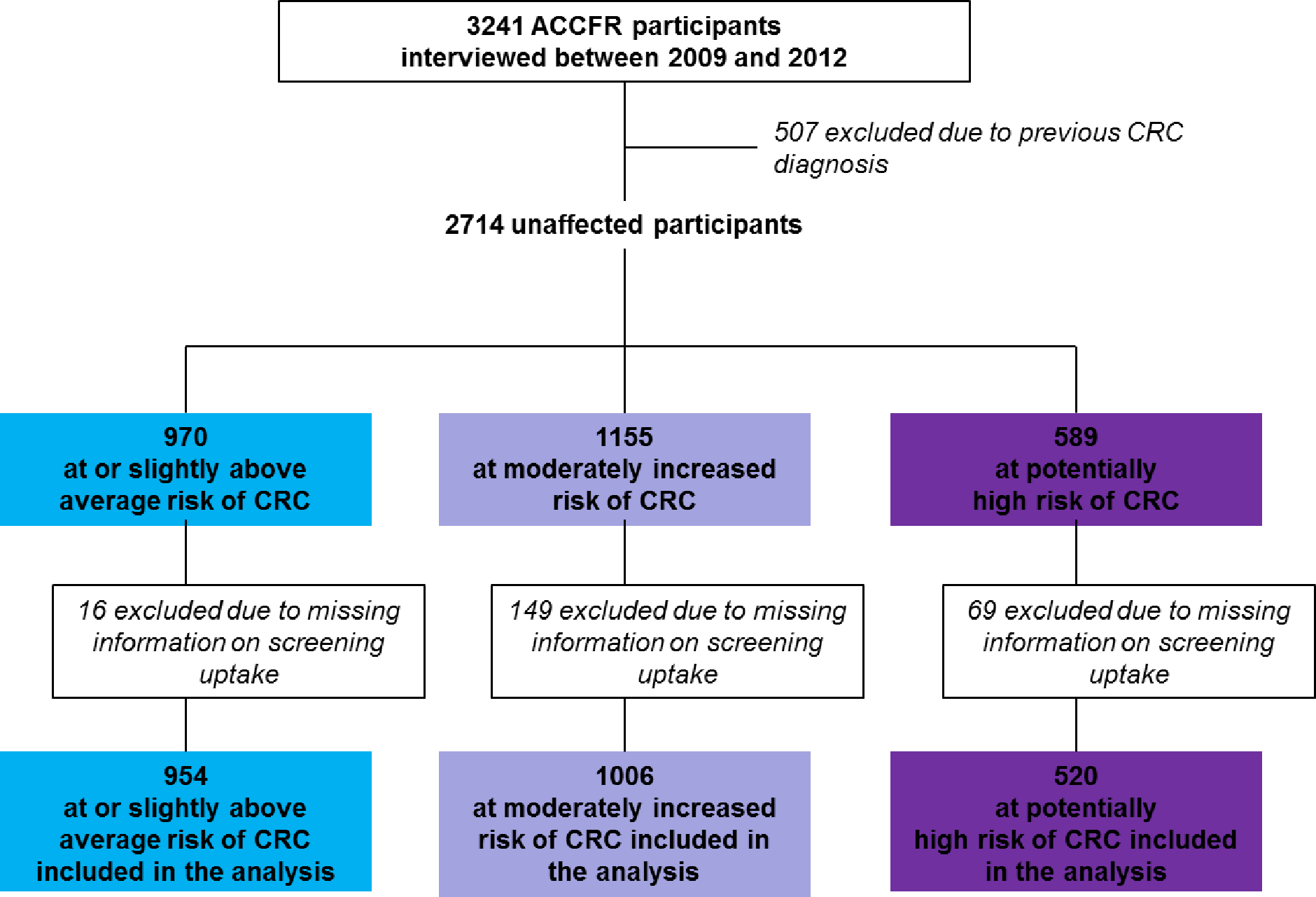

Supplement: S1 Fig — ACCFR, Australasian Colorectal Cancer Family Registry. (TIF) [file pmed.1002630.s002.tif]

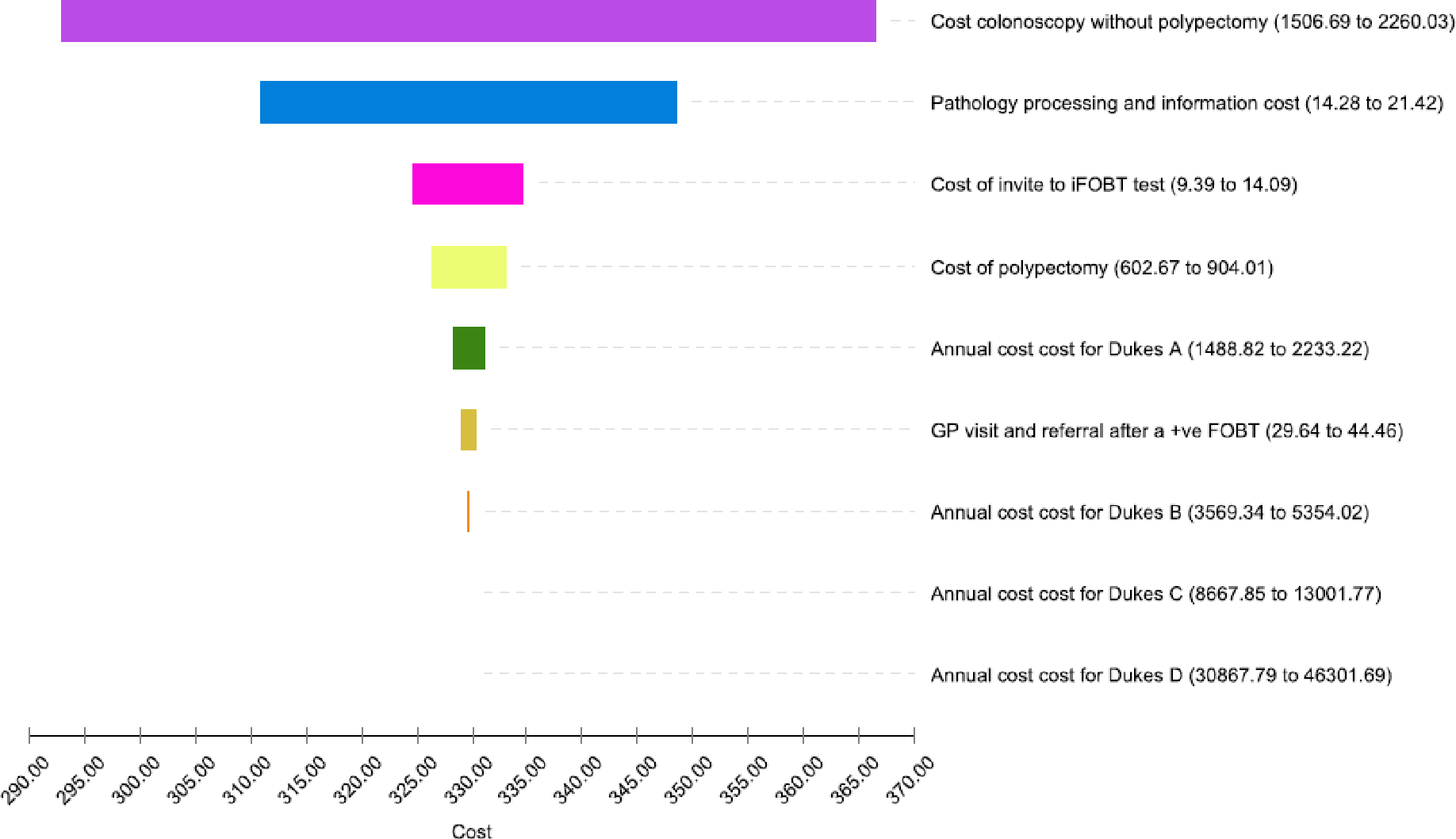

Supplement: S2 Fig — (TIF) [file pmed.1002630.s003.tif]

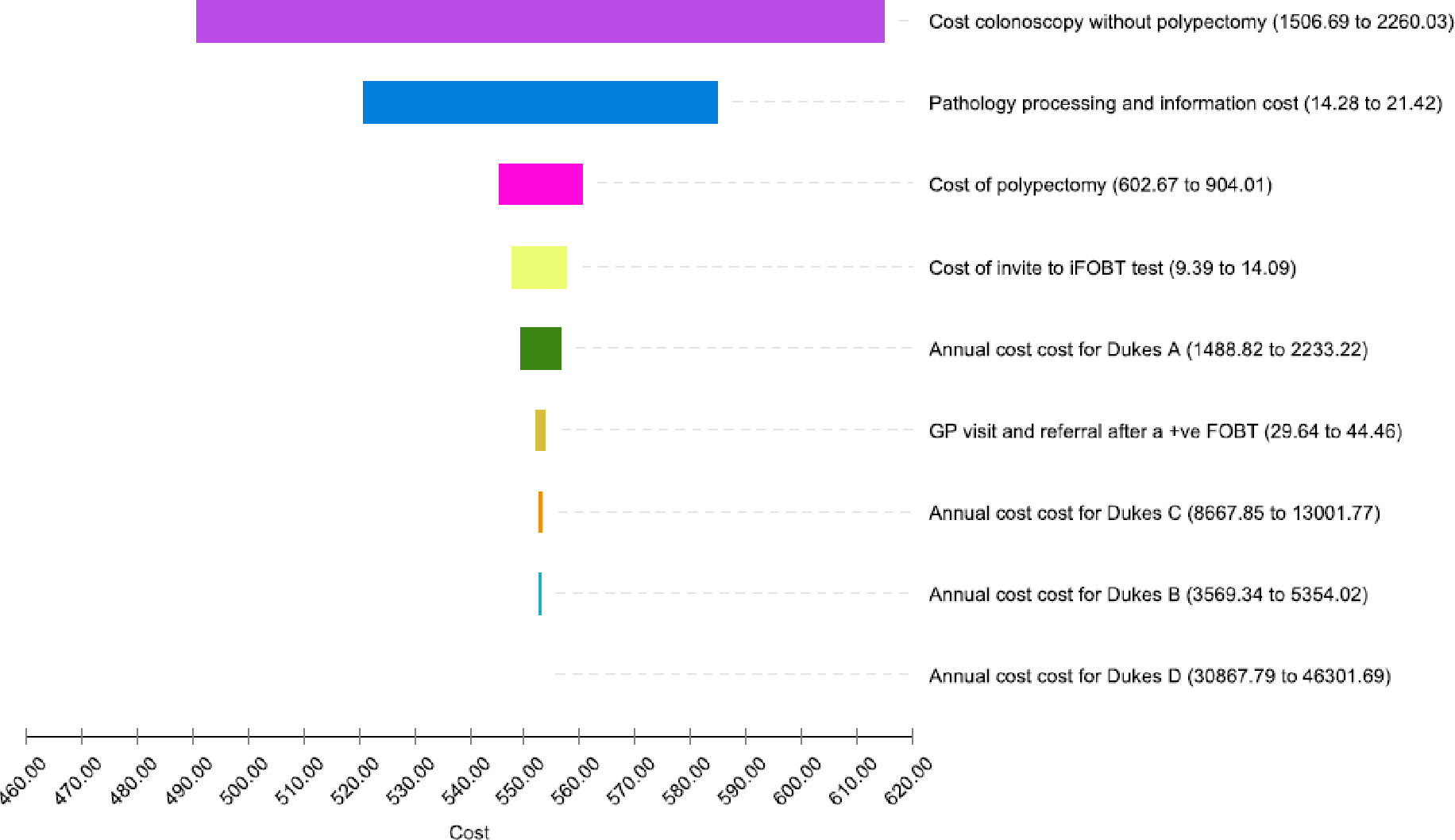

Supplement: S3 Fig — (TIF) [file pmed.1002630.s004.tif]

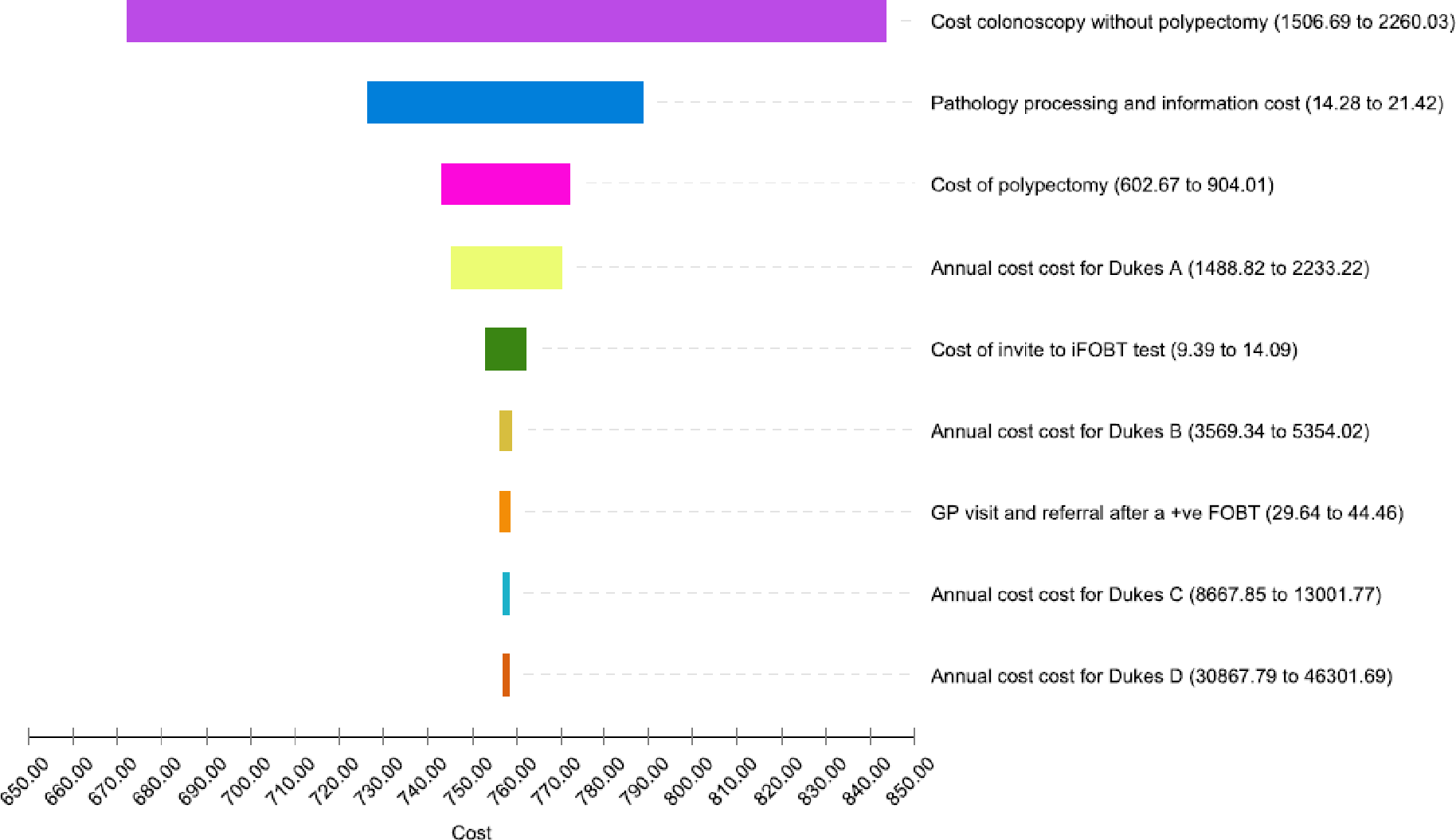

Supplement: S4 Fig — (TIF) [file pmed.1002630.s005.tif]

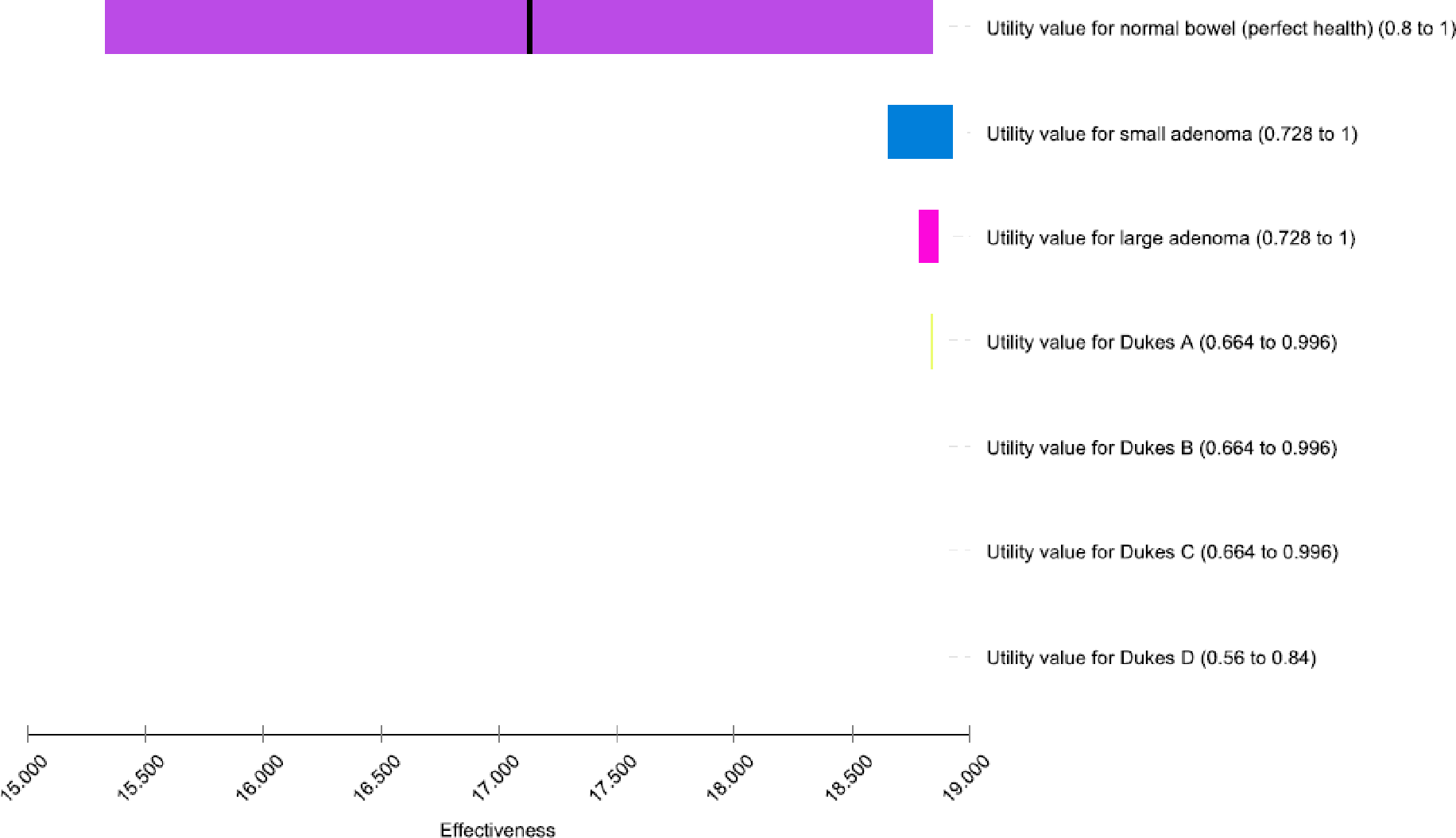

Supplement: S5 Fig — (TIF) [file pmed.1002630.s006.tif]

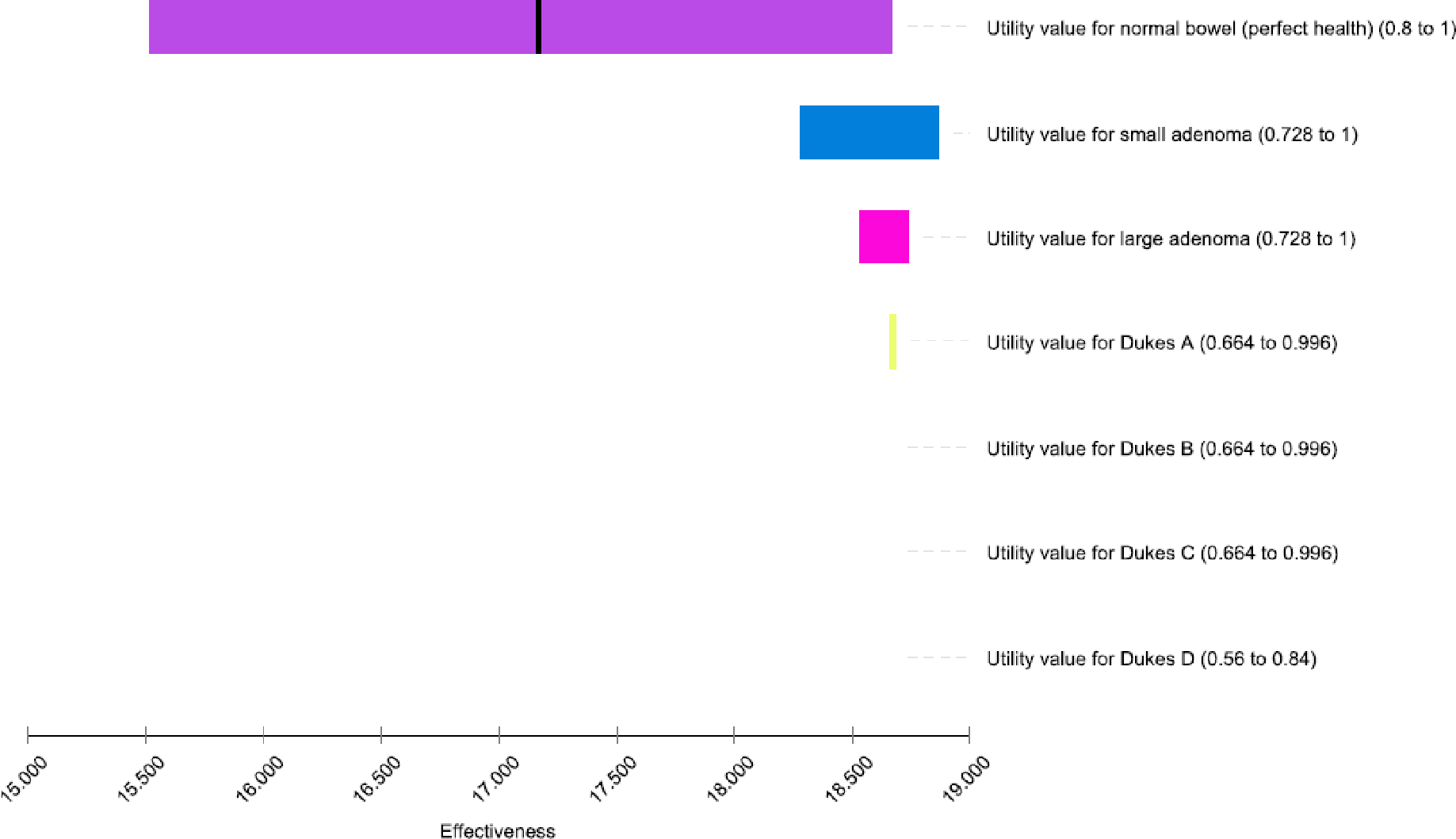

Supplement: S6 Fig — (TIF) [file pmed.1002630.s007.tif]

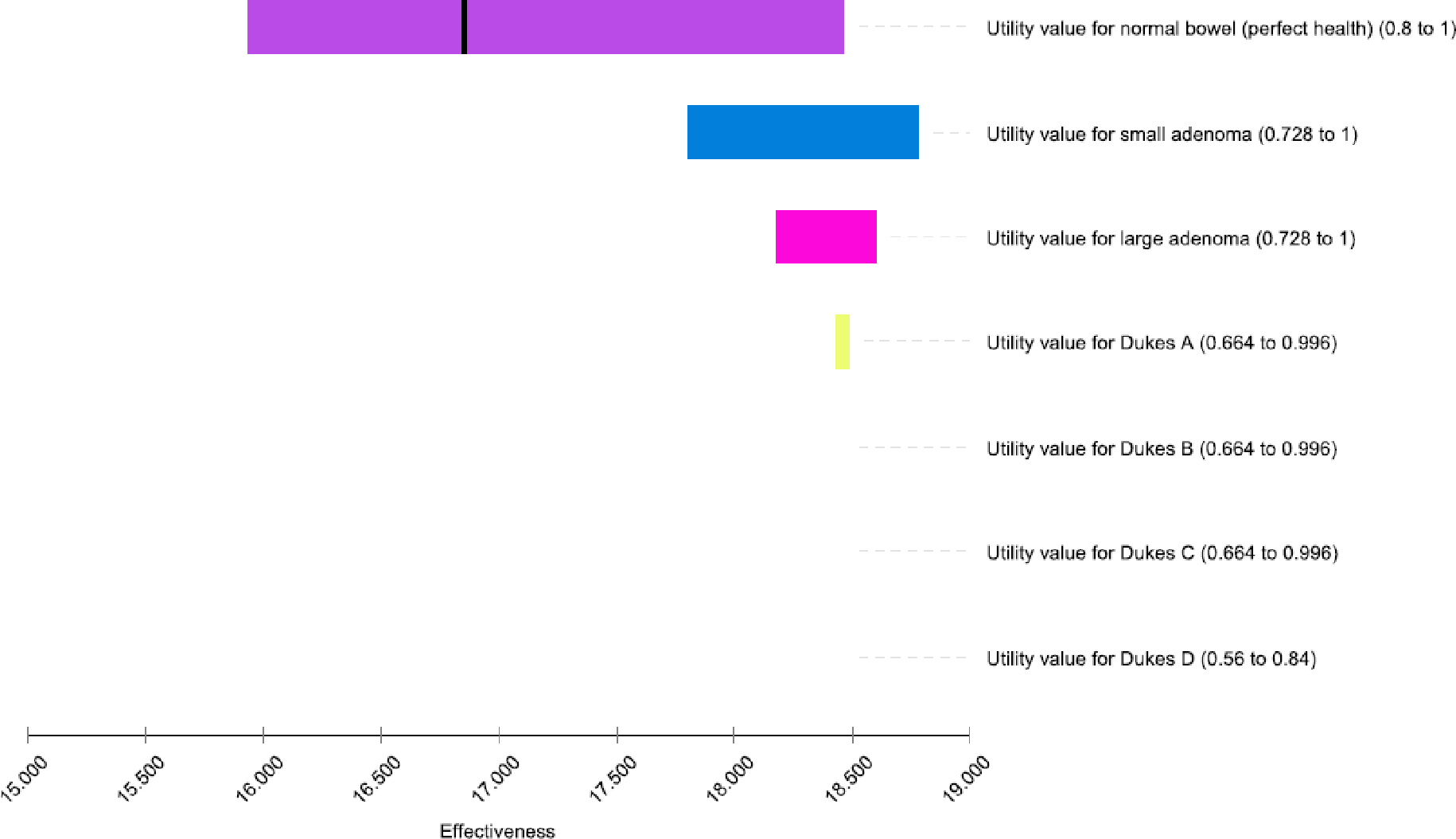

Supplement: S7 Fig — (TIF) [file pmed.1002630.s008.tif]
