# Supplementary material for: Family history–based colorectal cancer screening in Australia: A modelling study of the costs, benefits, and harms of different participation scenarios
Source: PLoS Med. 2018 Aug 16;15(8):e1002630. doi: 10.1371/journal.pmed.1002630 (PMC6095490; doi:10.1371/journal.pmed.1002630)
Supplement: S1 Table — (DOCX) [file pmed.1002630.s009.docx]

**S1 Table.** Model parameters

| **Parameter** | **Values** | | | **Source** |
| --- | --- | --- | --- | --- |
| **Initial state probabilities, age 25 (%)** | **Risk category 1:** | **Risk category 2:** | **Risk category 3:** |  |
| Normal bowel | 99.56 | 98.83 | 95.64 | [1] |
| Adenoma < 1cm | 0.33 | 0.89 | 3.29 |  |
| Adenoma > 1cm | 0.09 | 0.25 | 0.93 |  |
| Dukes' A | 0.01 | 0.02 | 0.07 |  |
| Dukes' B | 0.00 | 0.01 | 0.03 |  |
| Dukes' C | 0.00 | 0.01 | 0.03 |  |
| Dukes' D | 0.00 | 0.00 | 0.00 |  |
| **Utility values*** | **Value** | **Worst-Case Value** | **Best-Case Value** |  |
| Normal bowel  Post treatment normal bowel | 1.00 | 0.80 | 1.00 | Assumption |
| Adenoma < 1cm  Surveillance adenoma < 1cm | 0.91 | 0.728 | 1.00 | [2] |
| Adenoma > 1cm  Surveillance adenoma > 1cm | 0.91 | 0.728 | 1.00 |  |
| Dukes' A  Treatment Dukes' A | 0.83 | 0.664 | 0.996 | [3] |
| Dukes' B  Treatment Dukes' B | 0.83 | 0.664 | 0.996 |  |
| Dukes' C  Treatment Dukes' C | 0.83 | 0.664 | 0.996 |  |
| Dukes' D  Treatment Dukes' D | 0.70 | 0.56 | 0.84 |  |
| Death (other causes)  Death CRC | 0.00 | 0.00 | 0.00 | [4] |
| **iFOBT screening characteristics (%)** | | | | |
| Sensitivity for polyps | 21.19 | | | [1] |
| Sensitivity for CRC | 83.40 | | | [5] |
| Specificity for CRC | 92.60 | | |  |
| **Colonoscopy characteristics (%)** | | | | |
| Sensitivity for CRC | 95.00 | | | [1] |
| Sensitivity for polyps | 85.00 | | | [6] |
| Specificity for CRC | 100.00 | | | [1] |
| Chance of perforation | 0.068 | | | [7,8] |
| Chance of bleed | 0.14 | | |  |
| Chance of death | 0.008 | | |  |
| **Participation to NBCSP (%)** | | | | |
| Participating in the NBCSP | 39.00 | | | [9] |
| Colonoscopy uptake after positive iFOBT | 85.00^ | | |  |
| **Costs (AU$)** | **Value** | **Worst-Case Value** | **Best-Case Value** |  |
| iFOBT invitation and test kit | 11.74 | 9.39 | 14.09 | [1] |
| iFOBT pathology | 17.85 | 14.28 | 21.42 | [10] |
| GP visit & referral | 37.05 | 29.64 | 44.46 | [10] |
| Colonoscopy | 1,883.36 | 1,506.69 | 2,260.03 | [11] |
| Colonoscopy with polypectomy | 2,636.70 | 2,109.36 | 3,164.04 |  |
| Treatment Dukes' A (annual) | 1,861.02 | 1,488.82 | 2,233.22 | [12] |
| Treatment Dukes' B (annual) | 4,461.68 | 3,569.34 | 5,354.02 |  |
| Treatment Dukes' C (annual) | 10,834.81 | 8,667.85 | 13,001.77 |  |
| Treatment Dukes' D (annual) | 38,584.74 | 30,867.79 | 46,301.69 |  |

* Utility values are on a scale from zero, referring to the absence of life (death), to one, which is regarded as perfect health.

^ An assumed 15% was added to the monitoring report number as return of NBCSP forms is not mandatory, there may be incomplete form return and incomplete data. Note, percentages may not sum to 100% due to rounding

**References**

1. Bishop J, Glass P, Tracey E, Handy M, Warner K, Makino K*, et al.* Health Economics Review of Bowel Cancer Screening in Australia Cancer Institute NSW; 2008.

2. Ness RM, Holmes AM, Klein R, Dittus R. Cost-utility of one-time colonoscopic screening for colorectal cancer at various ages. Am J Gastroenterol **2000**;95(7):1800-11 doi 10.1111/j.1572-0241.2000.02172.x.

3. Snowsill T, Huxley N, Hoyle M, Jones-Hughes T, Coelho H, Cooper C*, et al.* A systematic review and economic evaluation of diagnostic strategies for Lynch syndrome. Health Technol Assess **2014**;18(58):1-406 doi 10.3310/hta18580.

4. Australian Bureau of Statistics (2013). 3101.0 - Australian Demographic Statistics. Available from: <http://www.abs.gov.au/ausstats/abs@.nsf/mf/3101.0>.

5. Australian Institute of Health and Welfare (2014). Analysis of bowel cancer outcomes for the National Bowel Cancer Screening Program. Cat. no. CAN 87. Canberra: AIHW.

6. NHMRC. National Health and Medical Research Council (NHMRC) 2005. Clinical practice guidelines for the prevention, early detection and management of colorectal cancer. Canberra NHMRC.

7. Rabeneck L, Paszat LF, Hilsden RJ, Saskin R, Leddin D, Grunfeld E*, et al.* Bleeding and perforation after outpatient colonoscopy and their risk factors in usual clinical practice. Gastroenterology **2008**;135(6):1899-906, 906 e1 doi 10.1053/j.gastro.2008.08.058.

8. Viiala CH, Zimmerman M, Cullen DJ, Hoffman NE. Complication rates of colonoscopy in an Australian teaching hospital environment. Intern Med J **2003**;33(8):355-9.

9. Australian Institute of Health and Welfare (2017). National Bowel Cancer Screening Program: monitoring report 2017. 103. Canberra: Cancer series.

10. Medicare Benefits Schedule (2016). The April 2016 Medicare Benefits Schedule. Available from: [http://www.mbsonline.gov.au/internet/mbsonline/publishing.nsf/Content /Downloads-201504](http://www.mbsonline.gov.au/internet/mbsonline/publishing.nsf/Content%20/Downloads-201504).

11. Independent Hospital Pricing Authority (2016). Australian Public Hospitals Cost Report 2013-2014 Round 18. Available from <https://www.ihpa.gov.au/publications/australian-public-hospitals-cost-report-2013-2014-round-18>.

12. Tran B, Keating CL, Ananda SS, Kosmider S, Jones I, Croxford M*, et al.* Preliminary analysis of the cost-effectiveness of the National Bowel Cancer Screening Program: demonstrating the potential value of comprehensive real world data. Intern Med J **2012**;42(7):794-800 doi 10.1111/j.1445-5994.2011.02585.x.
