# Supplementary material for: Family history–based colorectal cancer screening in Australia: A modelling study of the costs, benefits, and harms of different participation scenarios
Source: PLoS Med. 2018 Aug 16;15(8):e1002630. doi: 10.1371/journal.pmed.1002630 (PMC6095490; doi:10.1371/journal.pmed.1002630)
Supplement: S2 Table — (DOCX) [file pmed.1002630.s010.docx]

**S2 Table.** Incremental incidence for risk category 1, by age group

| **Variable** | **25-29** | **30-34** | **35-39** | **40-44** | **45-49** | **50-54** | **55-59** | **60-64** | **65-69** | **70-74** | **75-79** | **80-84** | **85+** |
| --- | --- | --- | --- | --- | --- | --- | --- | --- | --- | --- | --- | --- | --- |
| Normal | 0.9996 | 0.9993 | 0.9993 | 0.9989 | 0.9988 | 0.9968 | 0.9966 | 0.9963 | 0.9960 | 0.9958 | 0.9956 | 0.9951 | 0.9951 |
| Adenoma < 10 mm | 0.0003 | 0.0006 | 0.0006 | 0.0008 | 0.0008 | 0.0024 | 0.0024 | 0.0025 | 0.0024 | 0.0024 | 0.0024 | 0.0026 | 0.0026 |
| Adenoma > 10mm | 0.0001 | 0.0002 | 0.0002 | 0.0002 | 0.0002 | 0.0007 | 0.0007 | 0.0008 | 0.0008 | 0.0009 | 0.0009 | 0.0010 | 0.0010 |
| Dukes' A | 0.0000 | 0.0000 | 0.0000 | 0.0000 | 0.0001 | 0.0001 | 0.0001 | 0.0002 | 0.0004 | 0.0005 | 0.0006 | 0.0007 | 0.0006 |
| Dukes' B | 0.0000 | 0.0000 | 0.0000 | 0.0000 | 0.0000 | 0.0000 | 0.0001 | 0.0001 | 0.0002 | 0.0002 | 0.0003 | 0.0003 | 0.0003 |
| Dukes' C | 0.0000 | 0.0000 | 0.0000 | 0.0000 | 0.0000 | 0.0000 | 0.0001 | 0.0001 | 0.0001 | 0.0002 | 0.0002 | 0.0003 | 0.0003 |
| Dukes' D | 0.0000 | 0.0000 | 0.0000 | 0.0000 | 0.0000 | 0.0000 | 0.0000 | 0.0000 | 0.0000 | 0.0001 | 0.0001 | 0.0001 | 0.0001 |
