# Supplementary material for: Family history–based colorectal cancer screening in Australia: A modelling study of the costs, benefits, and harms of different participation scenarios
Source: PLoS Med. 2018 Aug 16;15(8):e1002630. doi: 10.1371/journal.pmed.1002630 (PMC6095490; doi:10.1371/journal.pmed.1002630)
Supplement: S3 Table — (DOCX) [file pmed.1002630.s011.docx]

**S3 Table.** Incremental incidence for risk category 2, by age group

| **Variable** | **25-29** | **30-34** | **35-39** | **40-44** | **45-49** | **50-54** | **55-59** | **60-64** | **65-69** | **70-74** | **75-79** | **80-84** | **85+** |
| --- | --- | --- | --- | --- | --- | --- | --- | --- | --- | --- | --- | --- | --- |
| Normal | 0.9989 | 0.9980 | 0.9980 | 0.9969 | 0.9968 | 0.9914 | 0.9911 | 0.9902 | 0.9894 | 0.9888 | 0.9881 | 0.9870 | 0.9871 |
| Adenoma < 10 mm | 0.0009 | 0.0015 | 0.0015 | 0.0023 | 0.0022 | 0.0062 | 0.0061 | 0.0064 | 0.0061 | 0.0059 | 0.0057 | 0.0061 | 0.0061 |
| Adenoma > 10mm | 0.0002 | 0.0004 | 0.0004 | 0.0007 | 0.0007 | 0.0020 | 0.0021 | 0.0023 | 0.0026 | 0.0028 | 0.0030 | 0.0034 | 0.0034 |
| Dukes' A | 0.0000 | 0.0000 | 0.0000 | 0.0001 | 0.0001 | 0.0002 | 0.0004 | 0.0005 | 0.0010 | 0.0012 | 0.0015 | 0.0017 | 0.0017 |
| Dukes' B | 0.0000 | 0.0000 | 0.0000 | 0.0000 | 0.0001 | 0.0001 | 0.0002 | 0.0002 | 0.0005 | 0.0006 | 0.0007 | 0.0008 | 0.0008 |
| Dukes' C | 0.0000 | 0.0000 | 0.0000 | 0.0000 | 0.0001 | 0.0001 | 0.0002 | 0.0002 | 0.0004 | 0.0005 | 0.0006 | 0.0007 | 0.0007 |
| Dukes' D | 0.0000 | 0.0000 | 0.0000 | 0.0000 | 0.0000 | 0.0000 | 0.0001 | 0.0001 | 0.0001 | 0.0002 | 0.0002 | 0.0002 | 0.0002 |
