# Supplementary material for: Family history–based colorectal cancer screening in Australia: A modelling study of the costs, benefits, and harms of different participation scenarios
Source: PLoS Med. 2018 Aug 16;15(8):e1002630. doi: 10.1371/journal.pmed.1002630 (PMC6095490; doi:10.1371/journal.pmed.1002630)
Supplement: S4 Table — (DOCX) [file pmed.1002630.s012.docx]

**S4 Table.** Incremental incidence for risk category 3, by age group

| **Variable** | **25-29** | **30-34** | **35-39** | **40-44** | **45-49** | **50-54** | **55-59** | **60-64** | **65-69** | **70-74** | **75-79** | **80-84** | **85+** |
| --- | --- | --- | --- | --- | --- | --- | --- | --- | --- | --- | --- | --- | --- |
| Normal | 0.9957 | 0.9927 | 0.9926 | 0.9885 | 0.9881 | 0.9676 | 0.9664 | 0.9634 | 0.9601 | 0.9580 | 0.9555 | 0.9513 | 0.9515 |
| Adenoma < 10 mm | 0.0032 | 0.0056 | 0.0055 | 0.0082 | 0.0080 | 0.0216 | 0.0203 | 0.0206 | 0.0184 | 0.0177 | 0.0172 | 0.0184 | 0.0184 |
| Adenoma > 10mm | 0.0009 | 0.0016 | 0.0017 | 0.0026 | 0.0029 | 0.0091 | 0.0104 | 0.0121 | 0.0143 | 0.0151 | 0.0156 | 0.0172 | 0.0172 |
| Dukes' A | 0.0001 | 0.0001 | 0.0001 | 0.0003 | 0.0005 | 0.0009 | 0.0014 | 0.0019 | 0.0035 | 0.0045 | 0.0057 | 0.0063 | 0.0063 |
| Dukes' B | 0.0000 | 0.0000 | 0.0001 | 0.0001 | 0.0002 | 0.0004 | 0.0007 | 0.0009 | 0.0017 | 0.0022 | 0.0028 | 0.0031 | 0.0031 |
| Dukes' C | 0.0000 | 0.0000 | 0.0001 | 0.0001 | 0.0002 | 0.0004 | 0.0006 | 0.0008 | 0.0015 | 0.0019 | 0.0024 | 0.0027 | 0.0027 |
| Dukes' D | 0.0000 | 0.0000 | 0.0000 | 0.0000 | 0.0001 | 0.0001 | 0.0002 | 0.0003 | 0.0005 | 0.0006 | 0.0008 | 0.0009 | 0.0009 |
