# Supplementary material for: Family history–based colorectal cancer screening in Australia: A modelling study of the costs, benefits, and harms of different participation scenarios
Source: PLoS Med. 2018 Aug 16;15(8):e1002630. doi: 10.1371/journal.pmed.1002630 (PMC6095490; doi:10.1371/journal.pmed.1002630)
Supplement: S5 Table — (DOCX) [file pmed.1002630.s013.docx]

**S5 Table.** Cost outcomes from the microsimulation in US Dollar

| **Screening Scenario** | **Average lifetime cost (US$/per person)** | **ICER (US$/QALY)** |
| --- | --- | --- |
| **Risk category 1** | | |
| Baseline | 361.01 | 6,503 |
| Current | 251.33 | - |
| Aspirational | 417.35 | 10,087 |
| Complete | 536.16 | 12,293 |
| **Risk category 2** | | |
| Baseline | 874.74 | - |
| Aspirational | 1,157.26 | 14,811 |
| Complete | 1,561.73 | 18,562 |
| **Risk category 3** | | |
| Baseline | 6,044.92 | - |
| Aspirational | 18,154.64 | 108,472 |
| Complete | 24,878.59 | 145,058 |
