# Supplementary material for: Family history–based colorectal cancer screening in Australia: A modelling study of the costs, benefits, and harms of different participation scenarios
Source: PLoS Med. 2018 Aug 16;15(8):e1002630. doi: 10.1371/journal.pmed.1002630 (PMC6095490; doi:10.1371/journal.pmed.1002630)
Supplement: S6 Table — (DOCX) [file pmed.1002630.s014.docx]

**S6 Table.** Sensitivity analysis of cost variables for all risk categories

| **Variable** | **Risk category 1 (AU$)** | | | **Risk category 2 (AU$)** | | | **Risk category 3 (AU$)** | | |
| --- | --- | --- | --- | --- | --- | --- | --- | --- | --- |
|  | **Low** | **High** | **Spread** | **Low** | **High** | **Spread** | **Low** | **High** | **Spread** |
| NBCSP invite | 324.76 | 334.64 | 9.88 | 547.94 | 557.66 | 9.72 | 752.95 | 762.35 | 9.40 |
| Pathology | 310.86 | 348.55 | 37.69 | 520.90 | 584.70 | 63.81 | 726.78 | 788.53 | 61.75 |
| GP visit | 329.09 | 330.31 | 1.22 | 552.01 | 553.59 | 1.58 | 756.50 | 758.80 | 2.30 |
| Colonoscopy | 292.93 | 366.47 | 73.54 | 491.04 | 614.56 | 123.53 | 672.03 | 843.27 | 171.24 |
| Polypectomy | 326.35 | 333.05 | 6.70 | 545.51 | 560.09 | 14.58 | 743.19 | 772.12 | 28.93 |
| Dukes' stage A | 328.28 | 331.12 | 2.84 | 549.18 | 556.42 | 7.23 | 745.42 | 769.88 | 24.46 |
| Dukes' stage B | 329.69 | 329.72 | 0.03 | 552.61 | 552.99 | 0.37 | 756.32 | 758.98 | 2.66 |
| Dukes' stage C | 329.70 | 329.70 | 0.00 | 552.64 | 552.96 | 0.32 | 757.02 | 758.29 | 1.27 |
| Dukes' stage D | 329.70 | 329.70 | 0.00 | 552.80 | 552.80 | 0.00 | 757.12 | 758.18 | 1.06 |
