# Supplementary material for: Family history–based colorectal cancer screening in Australia: A modelling study of the costs, benefits, and harms of different participation scenarios
Source: PLoS Med. 2018 Aug 16;15(8):e1002630. doi: 10.1371/journal.pmed.1002630 (PMC6095490; doi:10.1371/journal.pmed.1002630)
Supplement: S7 Table — (DOCX) [file pmed.1002630.s015.docx]

**S7 Table.** Sensitivity analysis of utility values for all risk categories

| **Variable** | **Risk category 1 (QALYs)** | | | **Risk category 2 (QALYs)** | | | **Risk category 3 (QALYs)** | | |
| --- | --- | --- | --- | --- | --- | --- | --- | --- | --- |
|  | **Low** | **High** | **Spread** | **Low** | **High** | **Spread** | **Low** | **High** | **Spread** |
| Normal bowel | 15.330 | 18.838 | 3.508 | 15.519 | 18.671 | 3.152 | 15.933 | 18.458 | 2.525 |
| Adenoma <1cm | 18.657 | 18.927 | 0.269 | 18.284 | 18.863 | 0.579 | 17.805 | 18.781 | 0.976 |
| Adenoma >1cm | 18.784 | 18.864 | 0.080 | 18.533 | 18.740 | 0.206 | 18.179 | 18.596 | 0.417 |
| Dukes' stage A | 18.833 | 18.843 | 0.010 | 18.658 | 18.684 | 0.026 | 18.434 | 18.483 | 0.049 |
| Dukes' stage B | 18.838 | 18.838 | 0.000 | 18.671 | 18.671 | 0.000 | 18.458 | 18.459 | 0.001 |
| Dukes' stage C | 18.838 | 18.838 | 0.000 | 18.671 | 18.671 | 0.000 | 18.458 | 18.458 | 0.000 |
| Dukes' stage D | 18.838 | 18.838 | 0.000 | 18.671 | 18.671 | 0.000 | 18.458 | 18.458 | 0.000 |
