# Supplementary material for: Family history–based colorectal cancer screening in Australia: A modelling study of the costs, benefits, and harms of different participation scenarios
Source: PLoS Med. 2018 Aug 16;15(8):e1002630. doi: 10.1371/journal.pmed.1002630 (PMC6095490; doi:10.1371/journal.pmed.1002630)
Supplement: S8 Table — (DOCX) [file pmed.1002630.s016.docx]

**S8 Table.** Sensitivity analysis of relative risk factors for all strategies, and risk categories

| **Screening scenario** | **Less risk** | | **More risk** | |
| --- | --- | --- | --- | --- |
|  | **Cost (AU$)** | **Effectiveness (QALYs)** | **Cost (AU$)** | **Effectiveness (QALYs)** |
| **Risk category 1** | | | | |
| Baseline | 472.88 | 18.840 | 471.87 | 18.836 |
| **Risk category 2** | | | | |
| Baseline | 1,144.12 | 18.662 | 1,145.98 | 18.670 |
| **Risk category 3** | | | | |
| Baseline | 7,932.51 | 18.460 | 7,913.99 | 18.455 |
